# Critical role of Wnt/β-catenin signaling in driving epithelial ovarian cancer platinum resistance

**DOI:** 10.18632/oncotarget.4690

**Published:** 2015-06-29

**Authors:** Anil Belur Nagaraj, Peronne Joseph, Olga Kovalenko, Sareena Singh, Amy Armstrong, Raymond Redline, Kimberly Resnick, Kristine Zanotti, Steven Waggoner, Analisa DiFeo

**Affiliations:** ^1^ Case Comprehensive Cancer Center, Case Western Reserve University, Cleveland, OH, USA; ^2^ Department of Gynecology, Division of Gynecological Oncology, University Hospital Case Medical Center, Cleveland, OH, USA; ^3^ Department of Pathology, University Hospital Case Medical Center, Cleveland, OH, USA

**Keywords:** patient derived xenograft, platinum resistance, tumor spheres, cancer initiating cells, β-catenin

## Abstract

Resistance to platinum-based chemotherapy is the major barrier to treating epithelial ovarian cancer. To improve patient outcomes, it is critical to identify the underlying mechanisms that promote platinum resistance. Emerging evidence supports the concept that platinum-based therapies are able to eliminate the bulk of differentiated cancer cells, but are unable to eliminate cancer initiating cells (CIC). To date, the relevant pathways that regulate ovarian CICs remain elusive. Several correlative studies have shown that Wnt/β-catenin pathway activation is associated with poor outcomes in patients with high-grade serous ovarian cancer (HGSOC). However, the functional relevance of these findings remain to be delineated. We have uncovered that Wnt/β-catenin pathway activation is a critical driver of HGSOC chemotherapy resistance, and targeted inhibition of this pathway, which eliminates CICs, represents a novel and effective treatment for chemoresistant HGSOC. Here we show that Wnt/β-catenin signaling is activated in ovarian CICs, and targeted inhibition of β-catenin potently sensitized cells to cisplatin and decreased CIC tumor sphere formation. Furthermore, the Wnt/β-catenin specific inhibitor iCG-001 potently sensitized cells to cisplatin and decreased stem-cell frequency in platinum resistant cells. Taken together, our data is the first report providing evidence that the Wnt/β-catenin signaling pathway maintains stem-like properties and drug resistance of primary HGSOC PDX derived platinum resistant models, and therapeutic targeting of this pathway with iCG-001/PRI-724, which has been shown to be well tolerated in Phase I trials, may be an effective treatment option.

## INTRODUCTION

Epithelial ovarian cancer [EOC] is the most lethal gynecologic malignancy and is the fifth leading cause of cancer deaths in women [1]. This year, it is estimated that approximately 22,000 women will be newly diagnosed and 14,200 will succumb to this lethal disease in the United States [1]. This poor overall survival is mainly due to late-stage diagnosis, disease recurrence and resistance to standard platinum-based chemotherapy. A large majority of EOC cases are high-grade serous cancers [HGSOC], which are initially highly sensitive to standard treatment of cytoreduction surgery and platinum-based chemotherapy, with response rate close to 85% [2]. In spite of such high initial response to platinum, the outcomes are poor, with 5-year survival rate of less than 30% with the majority of women who initially respond to platinum therapy relapsing [2]. Growing evidence supports the concept that platinum-based therapies are very efficient at eradicating differentiated cancer cells, but are unable to effectively eliminate CICs [3]. These rare populations of stem-like cells are characterized by their ability to self-renew indefinitely in an undifferentiated state. Furthermore, they result in differentiated progeny of cells with high proliferative and invasive capacity, thus driving the expansion of drug-resistant tumors [3-6].

Cisplatin treatment of ovarian tumors is associated with an increase in stem cell markers in the residual tumors, and accordingly, ovarian tumors that are chemo-resistant are reported to have increased expression of CIC markers [3, 6-9]. Even though numerous ovarian CIC markers have been identified, the signaling pathways regulating this phenotype are not well-understood [10]. In-depth characterization of the pathways that contribute to the emergence and maintenance of CICs in ovarian cancer is necessary in order to identify novel ways of targeting and eradicating CICs.

Canonical Wnt signaling, mediated through β-catenin, is identified as a critical regulator of chemo-resistance and CIC phenotype in many tumors, and inhibition of Wnt/β-catenin signaling is reported to increase sensitivity to chemotherapeutic agents in cancers [11-14]. However, the functional relevance of the Wnt/β-catenin pathway in regulating chemo-resistance and CIC phenotype in ovarian tumors is poorly understood. Unlike in other tumors, mutations in key signaling components of the canonical Wnt pathway are rare in HGSOC. However, several lines of evidence have implicated the potential activation of the Wnt pathway in these tumors, including (i) increased expression of β-catenin protein in HGSOC tumors [15, 16] (ii) correlation between β-catenin expression and poor survival in HGSOC and (iii) high expression of several Wnt ligands has been reported to be present in malignant ascites [17-19]. Most recently, a clustering analysis of the ovarian cancer gene expression data set of The Cancer Genome Atlas (TCGA) has identified altered Wnt/β-catenin components in tumors with poor prognosis, suggesting possible regulatory functions of Wnt/β-catenin in ovarian cancer [20]. In addition, gene expression profiling of chemo-resistant ovarian CICs has revealed up-regulation of several pathways including Wnt/β-catenin, and has determined an increase in CIC marker expression in these cells [21]. In sum, there have been numerous clinical correlations highlighting the association between disrupted Wnt/β-catenin signaling and patient outcome, but the functional relevance of this pathway in HGSOC remains to be delineated. In this study, we have directly investigated the role of Wnt/β-catenin signaling in the regulation of platinum resistance and stem-like properties in primary HGSOC patient-derived platinum-resistant models. We have identified Wnt/β-catenin as a novel driver of platinum resistance by maintaining stem-like properties in HGSOC. Targeted inhibition of this pathway overcomes platinum resistance by eradicating CICs.

## RESULTS

### Wnt/β-catenin signaling is up-regulated in platinum-resistant HGSOC tumors

**Figure 1:**
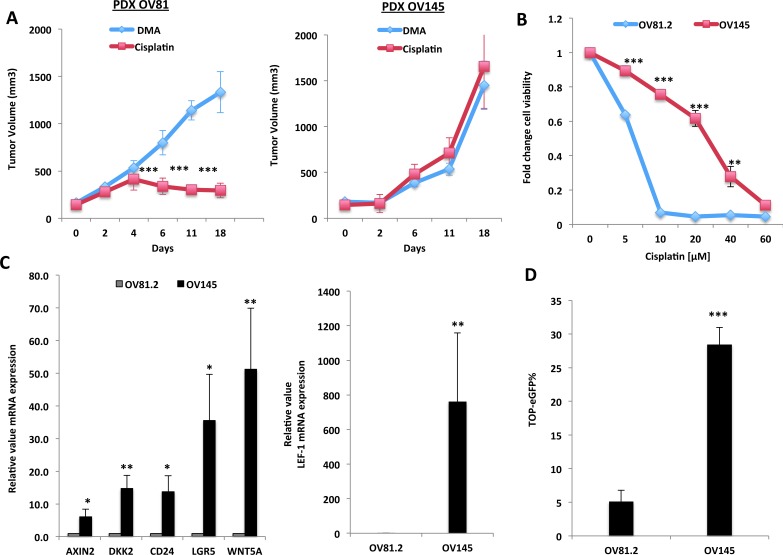
Wnt/β-catenin signaling is up-regulated in platinum-resistant HGSOC tumors **A.** Cisplatin treatment study in PDX tumors isolated from platinum-resistant patient (recurred within 6 months, right panel) and platinum sensitive (currently in remission >10 months, left panel). Mice were treated with 2.5mg/kg of cisplatin or PBS control after tumor implants reached ∼120mm^3^. **B.** 48h MTT assay with cisplatin confirming platinum resistance of OV145 *in-vitro*. **C.** Real-time PCR analysis showing increased mRNA expression of numerous Wnt/β-Catenin target genes in the platinum-resistant OV145 cell line, as compared to OV81 platinum-sensitive cell line. **D.** TOP-eGFP flow cytometry analysis showing increased TOP-eGFP% in platinum resistant OV145, as compared to platinum sensitive OV81.2.

In recent years, patient-derived xenograft (PDX) models have been utilized to conserve original tumor characteristics, such as heterogeneous histology, clinical biomolecular signature, malignant phenotypes and genotypes. Therefore, in order to assess the role of the Wnt/β-catenin pathway in regulating platinum resistance, we first examined the activity of this pathway in PDX models that display varying sensitivity to platinum-based therapies. In order to generate PDX models, chemotherapy-naive tumors were removed from patients diagnosed with HGSOC and ∼2mm^3^ tumor implants were grafted subcutaneously into nu/nu mice and propagated. Once excised, pathological comparison between the primary patient tumor and the PDX tumor were performed (Suppl. Figure 1). In order to assess platinum sensitivity, once the PDX tumors reached a size of ∼150mm^3^, the mice were treated with 2.5mg/kg of cisplatin or PBS control every other day. We found that the large majority of all tumors tested correlated with the patients platinum response. For example, the PDX tumor, OV145, which was generated from a patient who was deemed platinum-resistant given that she recurred within 6 months, was inherently resistant to platinum-based therapies compared to OV81, which was isolated from a patient who was platinum-sensitive (Figure 1A). In addition, the platinum therapy response of the cell lines generated from these tumors also correlated with patient platinum status *in-vitro* (Figure 1B). Upon confirmation of platinum sensitivity among these PDX tumors, we assessed the activity of the Wnt/β-catenin pathway. Numerous Wnt/β-catenin target genes, including Axin2, DKK2, Lef1, CD24 and Lgr5, were increased in the platinum-resistant OV145 cells [derived from OV145 platinum resistant PDX], as compared to the sensitive OV81.2 cells [derived from platinum sensitive OV81 PDX] (Figure 1C). Expression of the Wnt ligand WNT5A was very high in OV145, indicating that the Wnt pathway is highly activated in these platinum-resistant PDX-derived cells (Figure 1C). Furthermore, OV145 exhibited increased Wnt pathway activation, as shown through the utilization of an eGFP reporter fused TCF/LEF-1 Wnt/β-catenin reporter vector, which been shown to be an efficient indicator of β-catenin-regulated transcriptional activity in association with TCF7/LEF-1 [22, 23] (Figure 1D).

**Figure 2:**
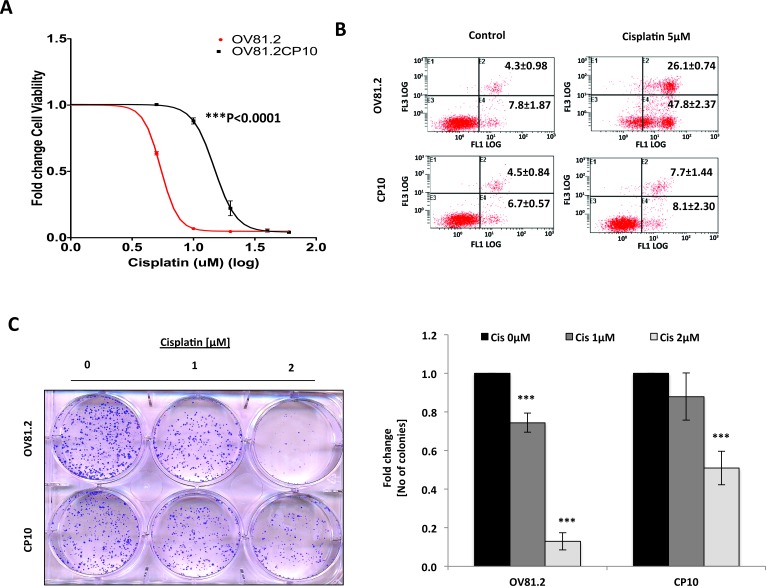
Generation of primary HGSOC PDX derived platinum-resistant model CP10 to understand mechanisms underlying platinum resistance in HGSOC **A.** 48h MTT assay showing 3-fold increase in IC50 value of cisplatin in CP10 platinum-resistant derivative of OV81.2 HGSOC cell line [48μM vs 14.7μM, p<0.0001]. **B.** Annexin-PI staining showing significantly less cell death in CP10, as compared to OV81.2 upon treatment with 5μM cisplatin for 72hrs. **C.** Clonogenics cell survival assay showing decreased sensitivity to platinum-therapy in CP10, as compared to OV81.2 upon treatment with 1μM and 2μM cisplatin for 7 days.

Next, in order to directly assess the effects of platinum therapy on the Wnt/β-catenin pathway, we examined whether long-term platinum therapy altered the activity of the Wnt/β-catenin pathway in OV81.2 primary tumor cells, derived from OV81 PDX. OV81.2 cells were propagated *in-vitro* in the presence of cisplatin in order to select for cells that have acquired platinum resistance [which is henceforth referred to as CP10]. Cellular viability assays revealed that CP10 was significantly more resistant to cisplatin, in comparison to the parental OV81.2 (Figure 2A). Furthermore, Annexin PI staining and clonogenic assays confirmed that these cells are highly resistant to platinum (Figure 2B and 2C). Annexin V staining, upon treatment with 5μM cisplatin, was ∼48% in the platinum-sensitive OV81.2, as compared to ∼8% in the platinum-resistant CP10, which was similar to the untreated cells (Figure 2B).

**Figure 3:**
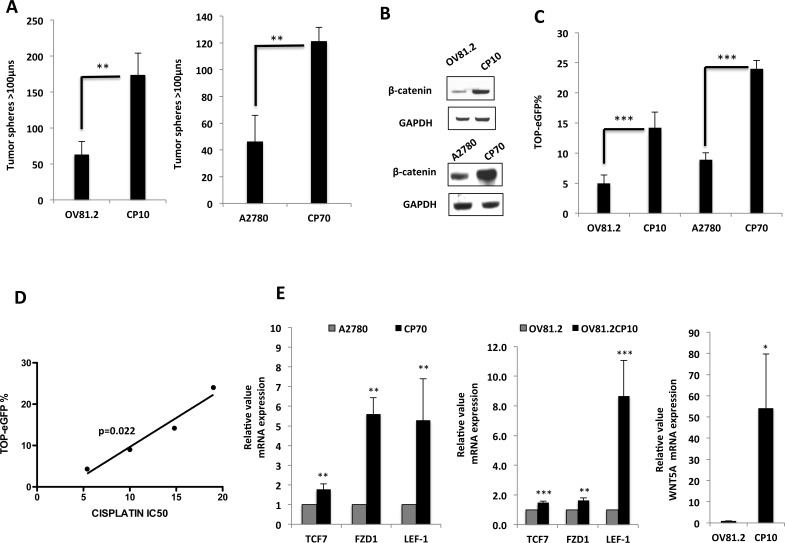
Long-term platinum therapy up-regulates the activity of the Wnt/β-catenin pathway in primary HGSOC **A.** 10×10 stitch imaging 10x and integrated analysis by METAMORPH software, showing increased tumor sphere formation in platinum-resistant CP10 and CP70 cells, as compared to matched platinum-sensitive OV81.2 and A2780, assessed on day 6. **B.** Western blots showing increased β-Catenin protein level in CP10 and CP70 platinum-resistant cells, as compared to their sensitive counterparts OV81.2 and A2780 respectively. **C.** Flow cytometry analysis showing increased TOP-eGFP reporter activity assessed 72hrs after transfection in CP10 and CP70, as compared to OV81.2 and A2780 respectively. **D.** Correlation analysis showing TOP-eGFP reporter activity is directly proportional to IC50 of Cisplatin in both platinum-sensitive and resistant cells. **E.** Real-time PCR showing increased mRNA expression of numerous Wnt/β-Catenin target genes in platinum-resistant cells. [* p<0.01, **p<0.001, ***p<0.0001 as compared to controls].

Given that emerging data suggest that platinum-resistance may emerge due to an enrichment in CICs [6], we next examined the ability of the newly generated primary platinum resistant cells to form non-adherent tumor spheres in serum-free stem-cell-selective conditions [24], which have been shown to enrich for CIC subpopulations and exhibit increased expression of CIC surface markers and oncogenes compared to adherent cultures. Interestingly, both CP10 and CP70, which is another established cisplatin resistant tumor cell line, derived from the A2780 ovarian tumor cell line [25], generated significantly more tumor spheres then their isogenic platinum-sensitive matched cell line (Figure 3A). Additionally, the platinum-resistant cells also had higher expression of well-known CIC markers like CD24, EpCAM and ALDH1, which are reported to be direct targets of Wnt/β-catenin signaling (Suppl. Figure 2).

In order to further define the molecular characteristics of these platinum-resistant stem-like cells, we examined the expression and functional activity of the Wnt/β-catenin signaling pathway, given that platinum-resistant PDX tumors revealed activation of this pathway compared to platinum-sensitive PDX tumors [18]. Interestingly, β-catenin protein level was higher in both CP10 and CP70 (Figure 3B), and this increased protein expression correlated with both CP10 and CP70 exhibiting >3-fold increase in TOP-eGFP Wnt reporter activity, as compared to their platinum-sensitive counterparts, showing that β-catenin-regulated transcriptional activity is increased in cisplatin-resistant ovarian tumor cells (Figure 3C). In addition, TOP-eGFP reporter activity in platinum-sensitive and resistant cells directly correlated with cisplatin IC50 values in these cells (Figure 3D). Further confirming up-regulated Wnt/β-catenin signaling in platinum resistant cells, we found that mRNA expression of Wnt/β-catenin transcriptional targets, TCF7, FZD1 and LEF-1, were up-regulated in platinum-resistant cells (Figure 3E). Also, the expression of WNT5A was very high in CP10 (Figure 3E), which was similar to the up-regulation observed in OV145 (Figure 1C), suggesting that the Wnt pathway is activated in both intrinsic (OV145) and acquired (CP10) models of platinum resistance in HGSOC.

### Wnt activation confers platinum resistance in primary ovarian tumor cells

**Figure 4:**
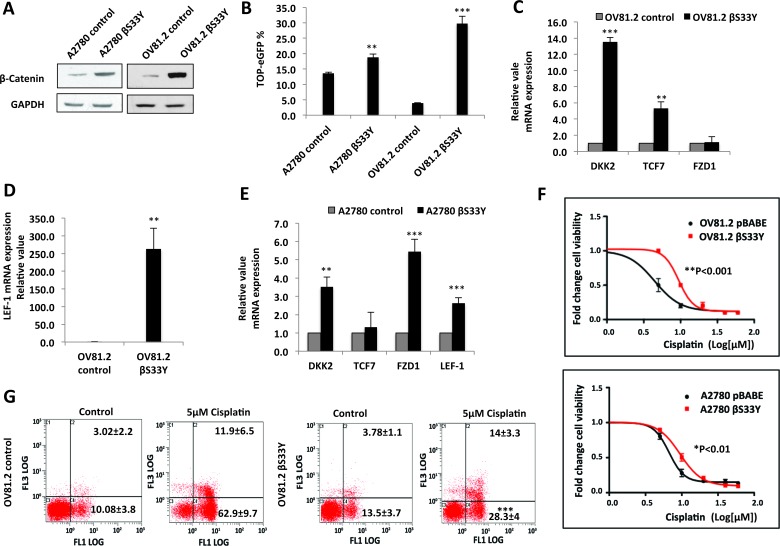
Stable overexpression of β-Catenin confers platinum resistance **A.** Western blot showing overexpression of β-CateninS33Y in A2780 and OV81.2. **B.** Flow cytometry analysis showing increased TOP-eGFP reporter activity, assessed 72hrs after transfection in A2780 βS33Y and OV81.2 βS33Y stable cell lines, as compared to their corresponding controls. **C.**, **D.** and **E.** Real-time PCR showing increased mRNA expression of Wnt/β-catenin transcriptional targets upon β-CateninS33Y overexpression. **F.** 24h MTT assay showing increased cisplatin IC50 in A2780 βS33Y [6.0μM to 9.5μM, p<0.01] and OV81.2 βS33Y [4.0μM to 9.7μM, p<0.001] stable cell lines. **G.** Annexin-PI staining showing a 2-fold decreased cell death in OV81.2 βS33Y cells, as compared to OV81.2 control cells, upon treatment with 5μM cisplatin for 72hrs (p<0.0001).

To assess whether Wnt/β-catenin pathway activation can directly render cells resistant to platinum, we overexpressed stabilized β-catenin S33Y [hereby referred to as βS33Y] in the platinum-sensitive A2780 and OV81.2 cells (Figure 4A). TOP-eGFP reporter activity was increased by βS33Y overexpression, indicating activated Wnt/β-catenin axis in these cells (Figure 4B). In addition, βS33Y overexpression increased the mRNA expression of Wnt/β-catenin transcriptional targets TCF7, FZD1, DKK1 and LEF-1 in both cell lines (Figure 4C-4E). Overexpression of βS33Y increased the IC50 of cisplatin by ∼2-fold in both A2780 (p<0.01) and OV81.2 (p<0.001) (Figure 4F). This increase was higher in OV81.2 βS33Y, which had higher TOP-eGFP reporter activity, as compared to A2780 βS33Y (Figure 4B), thus establishing a direct correlation between Wnt activation and resistance to cisplatin. In accordance with the cellular viability assays, Annexin-PI staining showed significantly decreased cell death in cisplatin treated OV81.2 βS33Y, as compared to the OV81.2 control (Figure 4G).

### β-catenin knockdown induces chemo-sensitivity in cisplatin-resistant ovarian tumor cells

**Figure 5:**
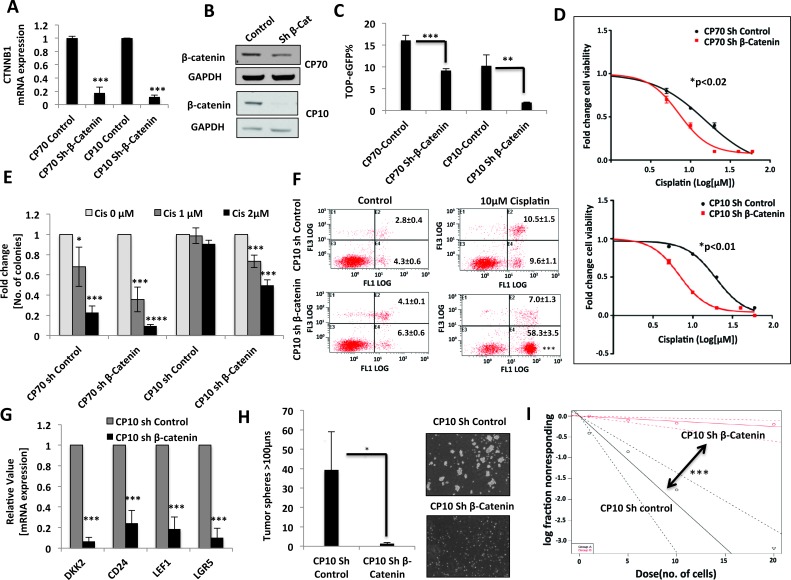
β-catenin knockdown induces chemo-sensitivity in platinum-resistant ovarian tumor cells **A.** and **B.** β-Catenin knockdown in platinum-resistant CP70 and CP10 cells, confirmed by β-Catenin [CTNNB1] mRNA down-regulation. **A.** and protein down-regulation **B.. C.** Flow cytometry analysis showing decreased TOP-eGFP reporter activity, assessed 72hrs after transfection in CP70 and CP10 sh β-Catenin stable cell lines, as compared to their corresponding shRNA control cell lines**. D.** 48h MTT assay showing decrease in cisplatin IC50 upon β-Catenin knockdown in platinum-resistant cells CP70 [15.4μM to 7.2μM, p<0.02] and CP10 [∼19.5μM to ∼6.7μM, p<0.01]. **E.** Clonogenics assay upon 7 days treatment with 1μM and 2 μM cisplatin, showing increased sensitivity to cisplatin upon β-catenin knockdown in both CP70 and CP10. **F.** Annexin-PI staining upon treatment with 10μM Cisplatin for 72hrs, showing ∼ 6 fold increase in cell death in CP10 upon β-catenin knockdown. **G.** Real-time PCR showing β-Catenin knockdown significantly decreases mRNA expression of Wnt/β-Catenin signaling-regulated stem cell markers in platinum-resistant CP10 cells. **H..**Representative 10x light microscopy images and 10×10 stitch imaging 10x and integrated analysis by METAMORPH software showing decreased tumor sphere formation in CP10 upon β-Catenin knockdown. **I.** Extreme Limiting dilution tumor sphere analysis [ELDA] showing decrease in stem cell frequency in CP10 upon β-Catenin knockdown.

Next, in order to directly ascetain whether the Wnt/β-catenin pathway is necessary for maintaining platinum resistance and for the maintenance of stem-like properties, we performed loss of function assays utilizing lentiviral shRNA mediated β-catenin knockdown [26]. β-catenin mRNA and protein expression were significantly downregulated in both the CP70 and CP10 platinum-resistant cells which stably expressed sh-β-catenin vector (Figure 5A and 5B), which correlated with a significant decrease in TOP-eGFP reporter activity (Figure 5C). β-catenin knockdown decreased the IC50 of cisplatin in both CP70 (2-fold, p<0.02) and CP10 (3-fold, p<0.01) (Figure 5D). The decrease in cisplatin IC50 was more robust in CP10 sh-β-catenin cells that had greater decrease in TOP-eGFP activity (Figure 5C), highlighting the direct correlation between Wnt activity and resistance to cisplatin. In addition, clonogenic cellular survival assays further confirmed that β-catenin knockdown increases cisplatin sensitivity in both CP70 and CP10 (Figure 5E). Furthermore, annexin-PI staining showed a >3-fold increase in cell death upon cisplatin treatment in CP10 sh-β-catenin cells, as compared to CP10 sh-control (Figure 5F). Upon examination of the expression of several CIC markers that have also been shown to be direct targets of Wnt/β-catenin, we found that CD24, LEF-1 and LGR5 were significantly reduced upon β-catenin knockdown in CP10 primary platinum resistant cells (Figure 5G). Consistent with these findings, β-catenin-targeted inhibition also significantly diminished the emergence of tumor spheres (Figure 5H). In order to further evaluate the effect of β-catenin knockdown on stem cell frequency in platinum resistant CP10, we employed limiting dilution tumor sphere assay [LDA]. LDA tumor sphere assays are increasingly being utilized to quantify stem cell frequency in tumor cells plated at very low cell density on non-adherent plates in serum free stem cell selective culture conditions [27]. We found that β-catenin knockdown drastically reduced the stem cell frequency in CP10 by >16-fold, from ∼1:5 cells to ∼1:80 cells (Figure 5I), suggesting that β-catenin is critical for maintaining stem-like properties in primary HGSOC platinum-resistant cells.

### β-catenin drives platinum resistance in ovarian CICs

**Figure 6:**
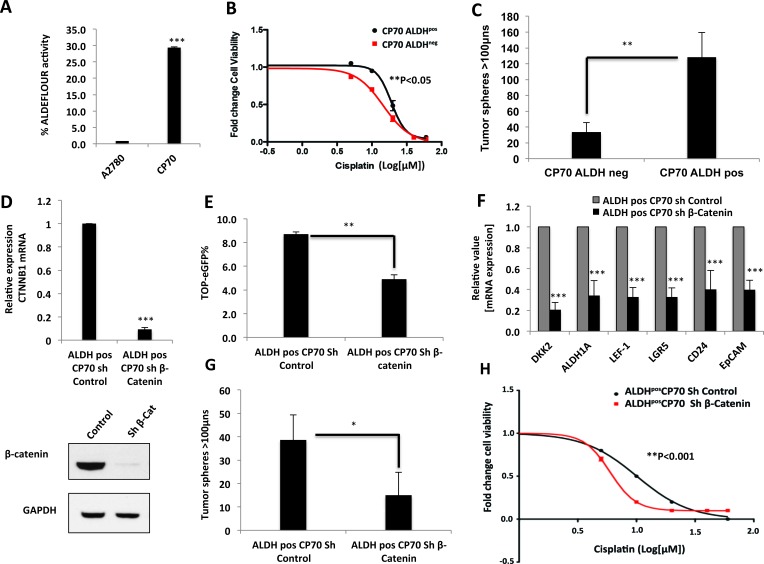
β-Catenin drives platinum resistance in ovarian cancer CICs **A.** ALDEFLOUR flow cytometry assay showing increased ALDH activity in platinum-resistant CP70 cells compared to platinum-sensitive A2780 cells. **B.** 48h MTT assay showing ALDH^pos^ CP70 cells are significantly more resistant to cisplatin (p<0.05). **C.** Tumor sphere formation is higher in ALDH^pos^ CP70 cells, as assessed by 10×10 stitch imaging 10x and integrated analysis by METAMORPH software. **D..**β-Catenin knockdown in platinum-resistant ALDH^pos^ CP70 cells confirmed by β-Catenin mRNA and protein down regulation. **E..**Flow cytometry analysis showing decreased TOP-eGFP reporter activity in ALDH^pos^ CP70 cells stably expressing sh-β-Catenin. **F.** β-Catenin knockdown decreases mRNA expression of Wnt/β-Catenin-regulated stem cell markers LEF-1, LGR5, CD24, EpCAM and ALDH1A1 in platinum-resistant ALDH^pos^ CP70. **G.** β-Catenin knockdown in platinum-resistant ALDH^pos^ CP70 cells decreases tumor sphere formation, as assessed by 10×10 stitch imaging 10x and integrated analysis by METAMORPH software. **H.** β-Catenin knockdown decreases cisplatin IC50 of platinum-resistant ALDH^pos^ CP70 cells, as assessed by 48h MTT assay (p<0.001).

Since β-catenin knockdown robustly decreased the stem cell frequency in platinum-resistant cells, we further studied whether β-catenin drives drug resistance in a cisplatin-resistant ovarian CIC model. Aldehyde dehydrogenase positive [ALDH^pos^] ovarian tumor cells have been identified as the CICs in ovarian cancer, and are reported to be chemo-resistant and associated with poor clinical outcomes [24, 28-32]. ALDH1A1 has recently been identified as a direct target of β-catenin, but the functional importance of this regulation is not yet understood [33]. Therefore, in order to study the role of β-catenin in regulating stem-like properties in ovarian CICs, we isolated CICs from the CP70 using ALDEFLOUR flow cytometry assay, which efficiently detects the ALDH1 isoform of aldehyde dehydrogenase [29, 33]. We found that CP70 cells had significantly higher ALDH1 activity, as compared to the cisplatin-sensitive A2780 cells (Figure 6A). We sorted ALDH^pos^ population from CP70 using ALDEFLOUR assay and observed that ALDH^pos^ CP70 cells were more resistant to platinum than ALDH^neg^ cells (p<0.05) (Figure 6B) and formed 4-fold more tumor spheres under stem cell culture conditions (Figure 6C). This tumor sphere-forming ability and ALDH1 activity was retained across multiple passages in stem cell culture conditions (Suppl. Figure 4) indicating the self-renewal ability of these cells. We next investigated whether β-catenin knockdown would abrogate the stem-like properties observed in the ALDH^pos^ population. Targeted shRNA of β-catenin knockdown (Figure 6D) resulted in decreased TOP-eGFP reporter activity (Figure 6E) and robustly decreased the expression of the numerous CIC markers such as ALDH1A1, CD24, EpCAM, LEF-1 and LGR5 (Figure 6F). Decrease in ALDH1A1 expression upon β-catenin knockdown is consistent with the recently reported finding that ALDH1A1 is a direct transcriptional target of β-catenin [33]. Furthermore, consistent with the decrease in CIC markers, β-catenin knockdown decreased the ability of ALDH^pos^ CICs to form tumor spheres (Figure 6G). Since decrease in stem-like population in tumor cells induces chemo-sensitivity, we next looked at the effect of β-catenin on cisplatin sensitivity of ALDH^pos^ CICs. β-catenin knockdown significantly decreased the IC50 for cisplatin in these cells (p<0.001) (Figure 6H), suggesting that β-catenin overcomes the platinum resistance of ALDH^pos^ CICs by decreasing stem-like properties of these cells.

### β-catenin maintains platinum resistance in ovarian cancer CICs

**Figure 7:**
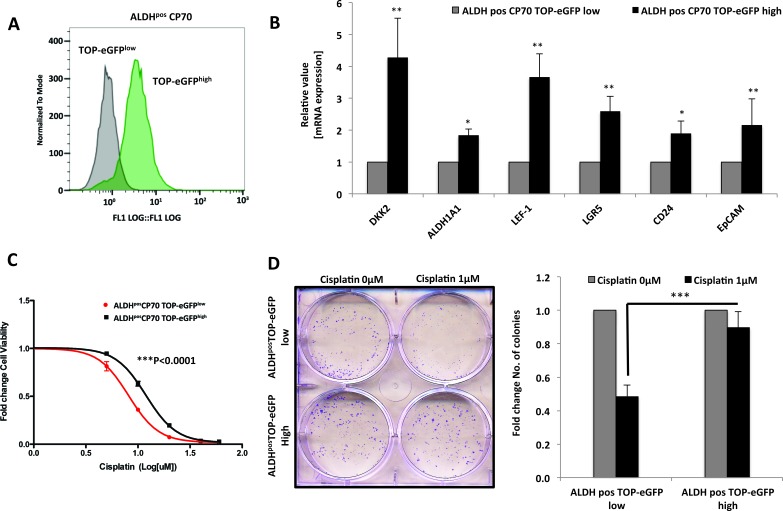
β-Catenin maintains platinum resistance in ovarian cancer CICs **A.** FACS sorting of TOP-eGFP^low^ and TOP-eGFP^high^ cells from ALDH^pos^ CP70 cells showing difference in eGFP expression between the sorted populations.**B.** Real time PCR showing higher expression of Wnt/β-Catenin regulated stem cell markers LEF-1, LGR5, CD24 and EpCAM in TOP-eGFP^high^ subpopulationof ALDH^pos^ CP70 cells **C.** and **D.** TOP-eGFP^high^ subpopulation of ALDH^pos^ CP70 cells are platinum-resistant when compared to TOP-eGFP^low^ subpopulationof ALDH^pos^ CP70 cells, as assessed by **D.** 48h MTT assay (p<0.0001), and exhibit increased survival in response to cisplatin treatment, as assessed by **E.** clonogenics assay on day 7 of cisplatin treatment.

To further delineate the regulation of platinum resistance in ovarian CICs by Wnt/β-catenin signaling, we sorted out TOP-eGFP ^high^ and TOP-eGFP ^low^ cell populations from ALDH^pos^ CICs by FACS and studied the differences in chemo-resistance between these two populations (Figure 7A). TOP-eGFP ^high^ALDH^pos^ cells had increased expression of numerous Wnt targets and CIC markers (Figure 7B). TOP-eGFP ^high^ALDH^pos^ cells were more resistant to cisplatin (p<0.0001) (Figure 7C) and exhibited increased survival in response to cisplatin treatment (Figure 7D). Collectively, these results indicate that within the ovarian cancer ALDH^pos^ CIC model, there exists a subpopulation of cells with high Wnt activity that have increased expression of CIC markers and are chemo-resistant, thus suggesting Wnt activity could be maintaining drug resistance in these CICs.

### Therapeutic targeting of β-catenin regulated transcriptional activity overcomes platinum resistance in chemo-resistant ovarian tumor cells and decreases stem cell frequency

**Figure 8:**
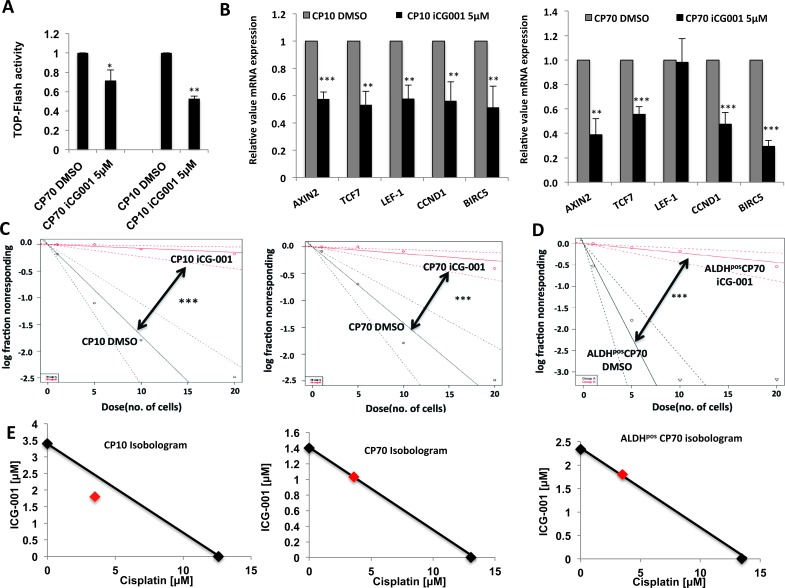
Therapeutic targeting of β-Catenin overcomes platinum resistance **A.** iCG-001 decreases TOP-Flash luciferase activity in CP70 and CP10. **B.** iCG-001 down-regulates Wnt/β-Catenin gene expression in platinum-resistant CP70 and CP10 cells. **C.** and **D.** limiting dilution tumor sphere assay with ELDA analysis showing decrease in stem cell frequency in CP10 and CP70 **C.** and ALDH^pos^ CP70 **D.** by 5μM iCG-001. **E.** Isobologram analysis of 48h MTT assay with cisplatin and iCG-001 combination, showing synergistic effect in CP10 (CI=0.8), additive effect in CP70 (CI=1) and ALDH^pos^ CP70 (CI=1).

Since our results showed that Wnt/β-catenin activity is a critical driver of chemo-resistance and stem-like properties in ovarian tumor cells, we next explored the possibility of therapeutic targeting of this pathway to overcome platinum resistance. Wnt/β-catenin inhibitors are reported to be effective in various tumor models and are being tested for their ability to target CICs [11, 14, 34]. The Wnt/β-catenin inhibitor iCG-001/PRI-724 specifically inhibits the β-catenin-regulated transcriptional axis important for stem cell maintenance and is currently being evaluated in phase 1 clinical trial in both solid tumors and hematological malignancies[11, 14, 35-37]. Prior to looking at the effects of iCG-001 on platinum resistance and stem-like properties, we first assessed its effects on the Wnt signaling pathway to confirm down-regulation of this pathway. We found that iCG-001 potently decreased TOP-Flash Wnt reporter activity (Figure 8A) and expression of Wnt/β-catenin targets in both CP70 and CP10 (Figure 8B) platinum-resistant cells.

We next assessed the effect of iCG-001 on stem-like properties in platinum-resistant CP10 and CP70 by LDA tumor sphere assays. iCG-001 robustly decreased stem cell frequency in CP10 and CP70 (Figure 8C). The decrease in stem cell frequency of CP10 was ∼23 fold (∼1:6 to ∼1:136) and in CP70 the decrease in stem-cell frequency was ∼11 fold (∼1:7 to 1:77), showing that targeting the β-catenin transcriptional axis greatly decreases stem-like properties in HGSOC. iCG-001 also decreased stem-cell frequency in ALDH^pos^ ovarian CICs by ∼20 fold (∼1:2.3 to 1:46), showing that therapeutic targeting of β-catenin could decrease stem-like properties in ovarian CICs (Figure 8D). Since a decrease in stem-like properties induces chemosensitivity, we next looked at the effect of combination treatment with iCG-001 and platinum across these models. Isobologram and combination index (CI) analysis of iCG-001 and cisplatin combination revealed that these drugs work in a synergistic manner in CP10 cells (CI=0.8), or in an additive fashion in CP70 (CI=1.01) and ALDH^pos^ ovarian CICs (CI=1.0) (Figure 8E). Collectively, these results show that therapeutic targeting of the β-catenin transcriptional axis by iCG-001 decreases stem-like properties and sensitizes platinum-resistant ovarian cancer cells.

## DISCUSSION

Current platinum-based treatment regimens in ovarian cancer are initially very effective at decreasing tumor burden because of reduction in tumor cells that are differentiated. However, CICs are known to be platinum-resistant and thus constitute one of the root causes of chemotherapy failure and tumor relapse. The absence of CIC-targeted therapies in ovarian cancer can be mainly attributed to a lack of in-depth understanding of the mechanisms driving and maintaining the stem-like properties in these cells. Signaling pathways like Notch have been implicated in regulating chemo-resistance in ovarian CICs, and targeting these cells by gamma secretase inhibitors [GSI] has been reported to increase response to platinum [21], highlighting the importance of understanding the underlying mechanisms driving ovarian CICs in order to appropriately target this distinct population of cells.

Numerous studies, including TCGA data analysis, has reported altered Wnt/β-catenin components in HGSOC, and correlative studies have shown that activation of this pathway is associated with poor patient outcomes [15, 17, 20]. In accordance with these findings, in our present study we have uncovered that activation of the Wnt/β-catenin pathway is associated with platinum-resistance in several *in vivo* and *in vitro* models. Specifically, we show that platinum-resistant PDX mouse models of HGSOC and primary platinum-resistant ovarian tumor cells have dramatically higher Wnt activity compared to matched platinum-sensitive cells. Our results support the involvement of Wnt/β-catenin signaling in both intrinsic and acquired platinum chemo-resistance. Wnt reporter activity was up-regulated in both the intrinsic model (OV145 PDX) and acquired model (OV81 PDX derived CP10) of platinum resistance. Up-regulation of Wnt ligand WNT5A was similar to both intrinsic and acquired models of platinum resistance, suggesting that the pathway is highly activated in both models of resistance.

Expression of the Wnt receptor FZD1 and the Wnt feedback component DKK2 correlated with Wnt/β-catenin activity and response to platinum in CP10 and CP70 models. Interestingly, the analysis of several publically available ovarian cancer cohorts revealed that FZD1 and DKK2 expression correlate with poor survival (Suppl. Figure 3) suggesting that high FZD1 and/or DKK2 expression in tumors could signify increased platinum-resistance and serve as a prognostic biomarker. Though these strong correlative findings highlight the clinical relevance of the Wnt/β-catenin pathway activation in ovarian cancer, there was a need to assess the functional significance of this pathway in order to determine whether it is a relevant therapeutic target in HGSOC, especially since mutations in Wnt/β-catenin pathway components are not known to be common in HGSOC. Therefore, we focused on the mechanistic role of Wnt/β-catenin pathway and identified canonical Wnt signaling mediated by β-catenin as a critical driver of platinum resistance in HGSOC.

Specific modulation of the Wnt/β-catenin pathway, either by β-catenin overexpression or knockdown, directly correlated with response to platinum-based therapy. β-catenin knockdown reversed platinum resistance and decreased stem cell frequency, which was associated with a decrease in the expression of stem cell markers like LEF-1 and LGR5. Intriguingly, these two markers were recently shown to be highly expressed in the ovarian stem cell niche [38], and LGR5 has been identified as a marker for the stem cell population in ovary [39]. Accordingly, the Wnt-specific inhibitor iCG-001 efficiently reversed platinum resistance and decreased tumor sphere formation in primary HGSOC cells. In further support of these results, it was recently reported that niclosamide decreased tumor sphere formation in ovarian tumors and Wnt activity was decreased in niclosamide-treated ovarian tumors [40]. However, this study did not investigate whether the Wnt/β-catenin pathway drives platinum resistance and stem-like properties. Additionally, niclosamide is known to inhibit multiple signaling pathways apart from Wnt [41], and hence, the effects observed in this study could be due to inhibition of multiple signaling pathways. Wnt-specific inhibitors, like iCG-001 used in our study, specifically inhibit the β-catenin-regulated transcriptional axis important for stem cell maintenance and is a more ideal drug to assess effects of Wnt-specific inhibition in ovarian CICs. In addition, PRI-724 (clinical compound generated from iCG-001) has shown an acceptable toxicity profile in Phase I clinical trials, and is currently being tested in several clinical trials. Thus, we uncovered that iCG-001 represent a clinically translatable drug which can: (1) sensitize both primary and immortalized ovarian cancers cell to cisplatin, (2) potently inhibit the Wnt signaling pathway, and (3) potentially eradicate ovarian CICs.

Intratumor heterogeneity in cancers is known to be associated with resistance to chemotherapy, and CICs are thought to play an important role in establishment and maintenance of this phenotype [42]. Ovarian tumors are known to have high degree of intratumor heterogeneity [43] and thus could contribute to chemotherapy resistance. Therefore, our findings showing that within platinum-resistant ALDH^pos^ ovarian CIC population there exists a sub-population of TOP-eGFP^high^ (Wnt-active) cells that are chemo-resistant and have higher expression of CIC markers, suggesting that Wnt activity could be critical in maintaining platinum resistance in ovarian CICs. Since ALDH1 is identified to be a direct target of β-catenin, and because we also saw a decrease in ALDH1 expression upon β-catenin knockdown, it could be speculated that the stem-like properties in ALDH^pos^ CICs in ovarian cancers could indeed be driven by β-catenin. Since primary HGSOC tumors have great complexity in terms of heterogeneity, TOP-eGFP sorting-based studies in primary HGSOC tumors are a more ideal way to understand the regulation of the phenotype and platinum resistance, in the context of tumor heterogeneity by Wnt/β-catenin signaling. In addition, TOP-eGFP based cell sorting in primary HGSOC tumors could be an efficient means of identifying gene signatures that reliably stratify Wnt^high^ and Wnt^low^ HGOSC tumors, thus enabling efficient Wnt/β-catenin-targeted therapy. Combination of cisplatin with CIC pathway-specific inhibitors in ovarian tumors could lead to personalized therapies targeting chemo-resistant subpopulations in ovarian tumors, thus forming an attractive approach for CIC-targeted therapy. Since there is extensive cross talk between the major signaling pathways regulating CIC phenotype the combination of CIC pathway-specific inhibitors with platinum-based regimens could greatly improve patient outcome in ovarian cancer.

## MATERIALS AND METHODS

### Cell culture and reagents

A2780 (cisplatin-sensitive) and CP70 (isogenic cisplatin-resistant to A2780) cells were obtained from Dr. Paul Modrich (Duke University). All cells used were cultivated in DMEM media supplemented with 10% FBS and 1% Penicillin-Streptomycin (recommended media). Cells were cultured in 10mm plates in a humidified atmosphere (5% CO_2_) at 37°C. At 70-90% confluence, trypsin (0.25%)/EDTA solution was used to detach the cells from the culture plate for passaging and used for further experiments until passage 20. Cisplatin was purchased from Mount Sinai Hospital Pharmacy. 10mM stock solutions of iCG-001 (Selleckchem) were prepared in DMSO (Fisher Scientific) and stored at −20°C. The OV81.2 cell line was generated from re-engrafted tumors that were derived from the ascites of a patient diagnosed with HGSOC. After successful engraftment of tumor cells into immunodeficient mice, tumor fragments were isolated and propagated on tissue culture plates *in-vitro*. To confirm tumorgenicity and conserved patient histology of these primary HGSOC cells, 500,000 cells were injected subcutaneously into immunodeficient mice and tumors that formed were subjected to histopathological analysis to allow for comparison to primary patient tumor. The platinum-resistant derivative of OV81.2, namely, CP10, was generated by propagating OV81.2 in the presence of 2.5μM cisplatin for 10 passages.

### Generation of stable cell lines

Several stable cell lines were generated for this study and the same protocol was followed as described below. TOP-eGFP lentiviral plasmid was acquired from Addgene (Addgene plasmid 24314 [22] deposited by Dr. Roel Nusse lab). β-catenin lentiviral shRNA plasmid (Addgene plasmid 18803 [26] deposited by Dr. Bob Weinberg's lab) and the corresponding pLKO.1 control plasmid (Addgene plasmid 8453 [44] deposited by Dr. Bob Weinberg's lab) were acquired from Addgene. For lentiviral transfection with the constructs, Lenti Starter Kit (System Biosciences) was used. Briefly, 3M 293T cells were plated in 10cm plate with antibiotic-free media. At 50-70% confluence, 2ug of plasmid and 10ug of pPACKH1-plasmid mix were co-transfected with Lipofectamine 2000 (Invitrogen) following manufacturer's protocol. 48 hours later, virus particles were harvested and precipitated. Target cells were transduced by plating 100,000 cells/well in a 6 well plate with virus particles and 4μg/mL polybrene and analyzed 72 hrs later. β-catenin-S33Y retroviral plasmid was a kind gift from Dr. Ken-Ichi Takemaru (Stony Brook University). For retroviral transfections, retrovirus was generated by co-transfection of the constructs with packaging plasmids into Phx cells. Target cells were transduced as described above.

### RNA extraction and real-time PCR

Total RNA was extracted using the Total RNA Purification Plus Kit (Norgen Biotek) according to manufacturer's instructions. For mRNA analysis, cDNA synthesis from 1μg of total RNA was done using the Transcriptor Universal cDNA Master kit (Roche). SYBR Green-based Real-time PCR was subsequently performed in triplicate using SYBR-Green master mix (Roche) on the Light Cycler 480 II Real-time PCR machine (Roche).

### Flow cytometry analysis and sorting

For ALDH based sorting, ALDH assay was done using the ALDEFLUOR kit as per the protocol instructions (Stem cell Technologies) and ALDH positive and negative cells were FACS sorted using BD FACS Aria II. For ALDH activity assessment, the same kit was used and the data was acquired with Coulter Epics XL machine. For TOP-eGFP sorting, eGFP low and high populations were sorted from TOP-eGFP transduced stable cell lines using BD FACS Aria II.

### Immunoblotting

Whole cell protein extracts were probed with antibodies against β-catenin (1:500) (Cell Signaling) and GAPDH (1:1000) (Santa Cruz) as previously described [45]. Membranes were exposed using LumiLight or LumiLight^plus^ (Roche) method following manufacturer's instructions.

### Cell viability assay

Cells were plated in 12-well plate at 50,000 cells/well and treated the next day with the corresponding drugs. After 24hrs or 48hrs, cells were then incubated with 3-(4,5-Dimethylthiazolyl) for 2hrs and absorbance was measured at 600nm. Isobologram analysis was done using GraphPad Prism software.

### Clonogenic assay

Cell survival was assessed through seeding 1000 cells/well in a 6-well plate and treated with indicated doses of cisplatin once every 3 days. On day 7, cells were fixed in a 10% acetic acid/10% methanol (in diH2O) solution and stained with 1% crystal violet (in methanol) after 7 days of growth. Colonies were counted using Image J.

### Annexin V/PI staining

Cells were plated 200,000 cells/well in a 6-well plate. The next day, cells were treated with drug and harvested 72 hours later. Annexin V/PI Staining was done using the FITC Annexin V Apoptosis Detection Kit II (BD Pharmigen). FACS data was acquired using the Coulter Epics XL machine.

### Tumor sphere formation assay

For tumor sphere formation assays, 1000 cells per ml were plated in 2ml in 6-well ultra-low attachment plate (Corning) in MammoCult medium (Stem cell Technologies) and after the indicated timepoint, 10×10 stitch imaging was done at 10x (100 random images acquired) using an Retiga Aqua Blue camera (Q Imaging, Vancouver, BC) connected to a Leica DMI6000 inverted microscope. Individual images were taken and then a composite image was generated using the scan slide function in Metamorph Imaging Software (Molecular Devices, Downington, PA). Subsequent integrated analysis also used Metamorph software. For limiting dilution sphere assays, BD FACS Aria II sorter was used to sort cells directly into 96-well ultra-low attachment plates in 200μl mammocult media per well (Corning). After 7 days, the number of wells with tumor spheres was counted and the data was analyzed by Extreme Limited Dilution Analysis [ELDA] platform to determine the stem cell frequency.

### TOP-Flash luciferase reporter assay

pTOP-Flash reporter construct stable cell lines were generated after lipofectamine 2000 [Life Technologies] based co-transfection of pTOP-flash construct with pBABE-puro plasmid [1:10 ratio] and puromycin-based selection. These were treated with iCG-001 and then cells were harvested after 8hrs and luciferase activities were analyzed using the Dual-Luciferase Reporter Assay System (Promega) with data normalization to the corresponding protein concentration

### Statistical analysis

Unless otherwise noted, data are presented as mean ± SD from three-independent experiments, and Student's *t*-test (two-tailed) was used to compare two groups (*P*<0.05 was considered significant) for independent samples.

## SUPPLEMENTARY MATERIALS, FIGURES



## References

[1] Siegel R, Ma J, Zou Z, Jemal A (2014). Cancer statistics, 2014. CA: a cancer journal for clinicians.

[2] Cooke SL, Brenton JD (2011). Evolution of platinum resistance in high-grade serous ovarian cancer. The Lancet Oncology.

[3] Ahmed N, Abubaker K, Findlay J, Quinn M (2013). Cancerous ovarian stem cells: obscure targets for therapy but relevant to chemoresistance. Journal of cellular biochemistry.

[4] Valent P, Bonnet D, De Maria R, Lapidot T, Copland M, Melo JV, Chomienne C, Ishikawa F, Schuringa JJ, Stassi G, Huntly B, Herrmann H, Soulier J, Roesch A, Schuurhuis GJ, Wohrer S (2012). Cancer stem cell definitions and terminology: the devil is in the details. Nature reviews Cancer.

[5] Kim CF, Dirks PB (2008). Cancer and stem cell biology: how tightly intertwined?. Cell stem cell.

[6] Steg AD, Bevis KS, Katre AA, Ziebarth A, Dobbin ZC, Alvarez RD, Zhang K, Conner M, Landen CN (2012). Stem cell pathways contribute to clinical chemoresistance in ovarian cancer. Clinical cancer research : an official journal of the American Association for Cancer Research.

[7] Ahmed N, Abubaker K, Findlay JK (2014). Ovarian cancer stem cells: Molecular concepts and relevance as therapeutic targets. Mol Aspects Med.

[8] Rizzo S, Hersey JM, Mellor P, Dai W, Santos-Silva A, Liber D, Luk L, Titley I, Carden CP, Box G, Hudson DL, Kaye SB, Brown R (2011). Ovarian cancer stem cell-like side populations are enriched following chemotherapy and overexpress EZH2. Molecular cancer therapeutics.

[9] Latifi A, Abubaker K, Castrechini N, Ward AC, Liongue C, Dobill F, Kumar J, Thompson EW, Quinn MA, Findlay JK, Ahmed N (2011). Cisplatin treatment of primary and metastatic epithelial ovarian carcinomas generates residual cells with mesenchymal stem cell-like profile. Journal of cellular biochemistry.

[10] Tomao F, Papa A, Strudel M, Rossi L, Lo Russo G, Benedetti Panici P, Ciabatta FR, Tomao S (2014). Investigating molecular profiles of ovarian cancer: an update on cancer stem cells. Journal of Cancer.

[11] Anastas JN, Moon RT (2013). WNT signalling pathways as therapeutic targets in cancer. Nature reviews Cancer.

[12] Heidel FH, Bullinger L, Feng Z, Wang Z, Neff TA, Stein L, Kalaitzidis D, Lane SW, Armstrong SA (2012). Genetic and pharmacologic inhibition of beta-catenin targets imatinib-resistant leukemia stem cells in CML. Cell stem cell.

[13] Yeung J, Esposito MT, Gandillet A, Zeisig BB, Griessinger E, Bonnet D, So CW (2010). beta-Catenin mediates the establishment and drug resistance of MLL leukemic stem cells. Cancer cell.

[14] Takahashi-Yanaga F, Kahn M (2010). Targeting Wnt signaling: can we safely eradicate cancer stem cells?. Clinical cancer research : an official journal of the American Association for Cancer Research.

[15] Barbolina MV, Burkhalter RJ, Stack MS (2011). Diverse mechanisms for activation of Wnt signalling in the ovarian tumour microenvironment. The Biochemical journal.

[16] Lee CM, Shvartsman H, Deavers MT, Wang SC, Xia W, Schmandt R, Bodurka DC, Atkinson EN, Malpica A, Gershenson DM, Hung MC, Lu KH (2003). beta-catenin nuclear localization is associated with grade in ovarian serous carcinoma. Gynecologic oncology.

[17] Gatcliffe TA, Monk BJ, Planutis K, Holcombe RF (2008). Wnt signaling in ovarian tumorigenesis. International journal of gynecological cancer : official journal of the International Gynecological Cancer Society.

[18] Arend RC, Londono-Joshi AI, Straughn JM (2013). and Buchsbaum DJ. The Wnt/beta-catenin pathway in ovarian cancer: a review. Gynecologic oncology.

[19] Bodnar L, Stanczak A, Cierniak S, Smoter M, Cichowicz M, Kozlowski W, Szczylik C, Wieczorek M, Lamparska-Przybysz M (2014). Wnt/beta-catenin pathway as a potential prognostic and predictive marker in patients with advanced ovarian cancer. Journal of ovarian research.

[20] Zhang W, Liu Y, Sun N, Wang D, Boyd-Kirkup J, Dou X, Han JD (2013). Integrating genomic, epigenomic, and transcriptomic features reveals modular signatures underlying poor prognosis in ovarian cancer. Cell reports.

[21] McAuliffe SM, Morgan SL, Wyant GA, Tran LT, Muto KW, Chen YS, Chin KT, Partridge JC, Poole BB, Cheng KH, Daggett J, Cullen K, Kantoff E, Hasselbatt K, Berkowitz J, Muto MG (2012). Targeting Notch, a key pathway for ovarian cancer stem cells, sensitizes tumors to platinum therapy. Proceedings of the National Academy of Sciences of the United States of America.

[22] Brugmann SA, Goodnough LH, Gregorieff A, Leucht P, ten Berge D, Fuerer C, Clevers H, Nusse R, Helms JA (2007). Wnt signaling mediates regional specification in the vertebrate face. Development.

[23] Staal FJ, Luis TC, Tiemessen MM (2008). WNT signalling in the immune system: WNT is spreading its wings. Nature reviews Immunology.

[24] Yasuda K, Torigoe T, Morita R, Kuroda T, Takahashi A, Matsuzaki J, Kochin V, Asanuma H, Hasegawa T, Saito T, Hirohashi Y, Sato N (2013). Ovarian cancer stem cells are enriched in side population and aldehyde dehydrogenase bright overlapping population. PloS one.

[25] Parker RJ, Eastman A, Bostick-Bruton F, Reed E (1991). Acquired cisplatin resistance in human ovarian cancer cells is associated with enhanced repair of cisplatin-DNA lesions and reduced drug accumulation. The Journal of clinical investigation.

[26] Onder TT, Gupta PB, Mani SA, Yang J, Lander ES, Weinberg RA (2008). Loss of E-cadherin promotes metastasis via multiple downstream transcriptional pathways. Cancer research.

[27] Rota LM, Lazzarino DA, Ziegler AN, LeRoith D, Wood TL (2012). Determining mammosphere-forming potential: application of the limiting dilution analysis. Journal of mammary gland biology and neoplasia.

[28] Januchowski R, Wojtowicz K, Zabel M (2013). The role of aldehyde dehydrogenase (ALDH) in cancer drug resistance. Biomedicine & pharmacotherapy = Biomedecine & pharmacotherapie.

[29] Kuroda T, Hirohashi Y, Torigoe T, Yasuda K, Takahashi A, Asanuma H, Morita R, Mariya T, Asano T, Mizuuchi M, Saito T, Sato N (2013). ALDH1-high ovarian cancer stem-like cells can be isolated from serous and clear cell adenocarcinoma cells, and ALDH1 high expression is associated with poor prognosis. PloS one.

[30] Landen CN, Goodman B, Katre AA, Steg AD, Nick AM, Stone RL, Miller LD, Mejia PV, Jennings NB, Gershenson DM, Bast RC, Coleman RL, Lopez-Berestein G, Sood AK (2010). Targeting aldehyde dehydrogenase cancer stem cells in ovarian cancer. Molecular cancer therapeutics.

[31] Wang YC, Yo YT, Lee HY, Liao YP, Chao TK, Su PH, Lai HC (2012). ALDH1-bright epithelial ovarian cancer cells are associated with CD44 expression, drug resistance, and poor clinical outcome. The American journal of pathology.

[32] Silva IA, Bai S, McLean K, Yang K, Griffith K, Thomas D, Ginestier C, Johnston C, Kueck A, Reynolds RK, Wicha MS, Buckanovich RJ (2011). Aldehyde dehydrogenase in combination with CD133 defines angiogenic ovarian cancer stem cells that portend poor patient survival. Cancer research.

[33] Condello S, Morgan CA, Nagdas S, Cao L, Turek J, Hurley TD, Matei D (2015). beta-Catenin-regulated ALDH1A1 is a target in ovarian cancer spheroids. Oncogene. Oncogene.

[34] Takebe N, Harris PJ, Warren RQ, Ivy SP (2011). Targeting cancer stem cells by inhibiting Wnt, Notch, and Hedgehog pathways. Nature reviews Clinical oncology.

[35] Emami KH, Nguyen C, Ma H, Kim DH, Jeong KW, Eguchi M, Moon RT, Teo JL, Kim HY, Moon SH, Ha JR, Kahn M (2004). A small molecule inhibitor of beta-catenin/CREB-binding protein transcription [corrected]. Proceedings of the National Academy of Sciences of the United States of America.

[36] Valkenburg KC, Graveel CR, Zylstra-Diegel CR, Zhong Z, Williams BO (2011). Wnt/beta-catenin Signaling in Normal and Cancer Stem Cells. Cancers.

[37] Huang SS, Clarke DC, Gosline SJ, Labadorf A, Chouinard CR, Gordon W, Lauffenburger DA, Fraenkel E (2013). Linking proteomic and transcriptional data through the interactome and epigenome reveals a map of oncogene-induced signaling. PLoS computational biology.

[38] Flesken-Nikitin A, Hwang CI, Cheng CY, Michurina TV, Enikolopov G, Nikitin AY (2013). Ovarian surface epithelium at the junction area contains a cancer-prone stem cell niche. Nature.

[39] Ng A, Tan S, Singh G, Rizk P, Swathi Y, Tan TZ, Huang RY (2014). Leushacke M and Barker N. Lgr5 marks stem/progenitor cells in ovary and tubal epithelia. Nat Cell Biol.

[40] Arend RC, Londono-Joshi AI, Samant RS, Li Y, Conner M, Hidalgo B, Alvarez RD, Landen CN, Straughn JM, Buchsbaum DJ (2014). Inhibition of Wnt/beta-catenin pathway by niclosamide: A therapeutic target for ovarian cancer. Gynecologic oncology.

[41] Pan JX, Ding K, Wang CY (2012). Niclosamide, an old antihelminthic agent, demonstrates antitumor activity by blocking multiple signaling pathways of cancer stem cells. Chinese journal of cancer.

[42] Michor F, Polyak K (2010). The origins and implications of intratumor heterogeneity. Cancer prevention research.

[43] Hoogstraat M, de Pagter MS, Cirkel GA, van Roosmalen MJ, Harkins TT, Duran K, Kreeftmeijer J, Renkens I, Witteveen PO, Lee CC, Nijman IJ, Guy T, van ‘t Slot R, Jonges TN, Lolkema MP, Koudijs MJ (2014). Genomic and transcriptomic plasticity in treatment-naive ovarian cancer. Genome research.

[44] Stewart SA, Dykxhoorn DM, Palliser D, Mizuno H, Yu EY, An DS, Sabatini DM, Chen IS, Hahn WC, Sharp PA, Weinberg RA, Novina CD (2003). Lentivirus-delivered stable gene silencing by RNAi in primary cells. Rna.

[45] Parikh A, Lee C, Peronne J, Marchini S, Baccarini A, Kolev V, Romualdi C, Fruscio R, Shah H, Wang F, Mullokandov G, Fishman D, D'Incalci M, Rahaman J, Kalir T, Redline RW (2014). microRNA-181a has a critical role in ovarian cancer progression through the regulation of the epithelial-mesenchymal transition. Nature communications.

