# Supplementary material for: Critical role of Wnt/β-catenin signaling in driving epithelial ovarian cancer platinum resistance
Source: Oncotarget. 2015 Jun 29;6(27):23720–34. doi: 10.18632/oncotarget.4690 (PMC4695147; doi:10.18632/oncotarget.4690)
Supplement: Supplementary file 1 [file oncotarget-06-23720-s001.pdf]

Critical Role of Wnt/β-catenin Signaling in Driving Epithelial Ovarian Cancer Platinum Resistance

Supplementary Material

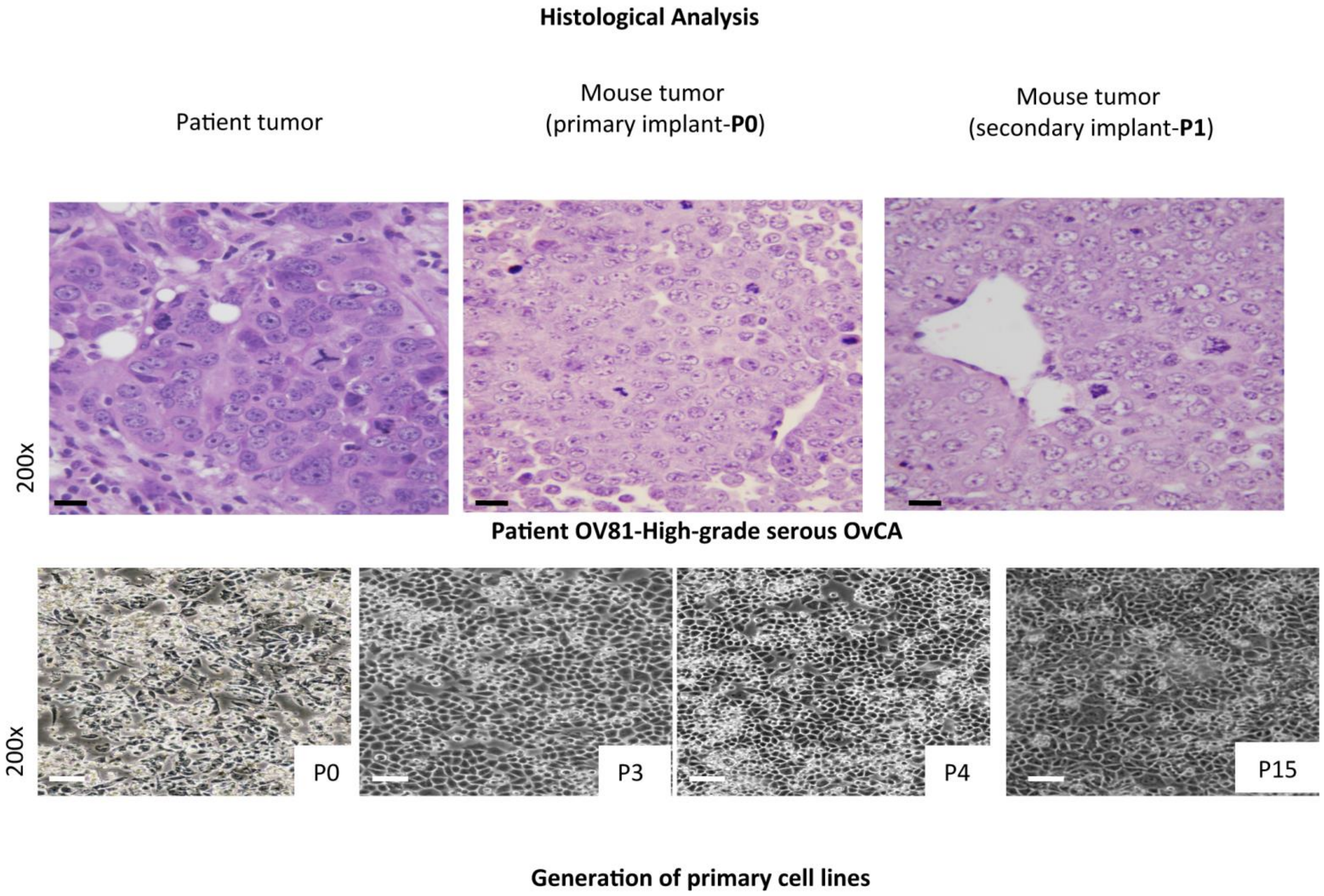

Supplementary Figure 1: Generating primary HGSOC PDX-derived OV81.2 and OV145 models

Sequential steps leading to the establishment of an ovarian cancer primary HGSOC Patient-Derived cell line: Briefly for the OV145 PDX model, HGSOC ovarian tumor fragments from operating room were directly xenografted in an immunodeficient mouse (Passage 0). After successful engraftment, new fragments were taken from the mouse hosted human tumor and xenografted again in multiple immunodeficient mice (Passage 1). A collection of fragments from the resulting tumors was directly re-engrafted in mice for expansion. The tumor fragments were subjected to histopathological analysis, comparing the fragments to the patient tumor. The generation of the OV81.2 model was similar to OV145, but rather than tumor fragments being injected into mice, the ascites from a patient diagnosed with HGSOC was injected into nude mice. After 2 passages *in-vivo*, the cells were propagated on tissue culture plates *in-vitro* to generate OV81.2 and OV145 cell lines.

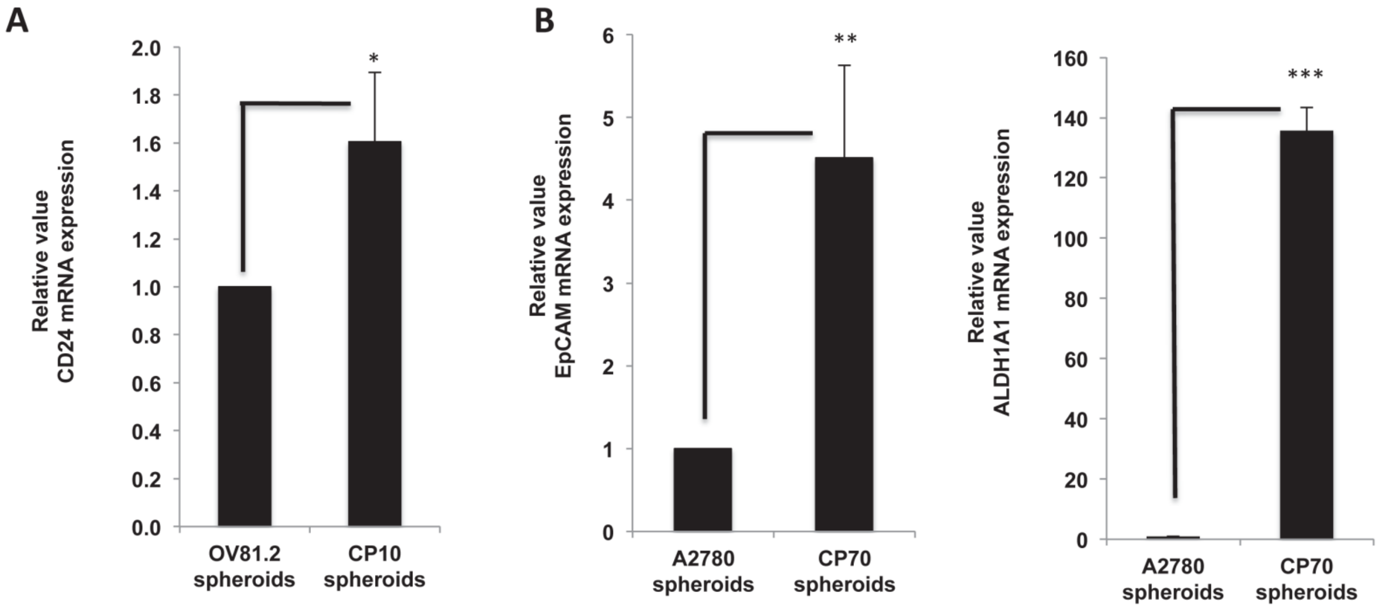

Supplementary Figure 2: Wnt/β-catenin regulated CIC marker expression is higher in platinum-resistant tumor spheres

Real-time PCR analysis showing increased expression of Wnt/β-catenin-regulated CIC markers like CD24 in CP10 (A), EpCAM and ALDH1A1 in CP70 (B)

A. FZD1 mRNA expression and survival in epithelial ovarian cancer

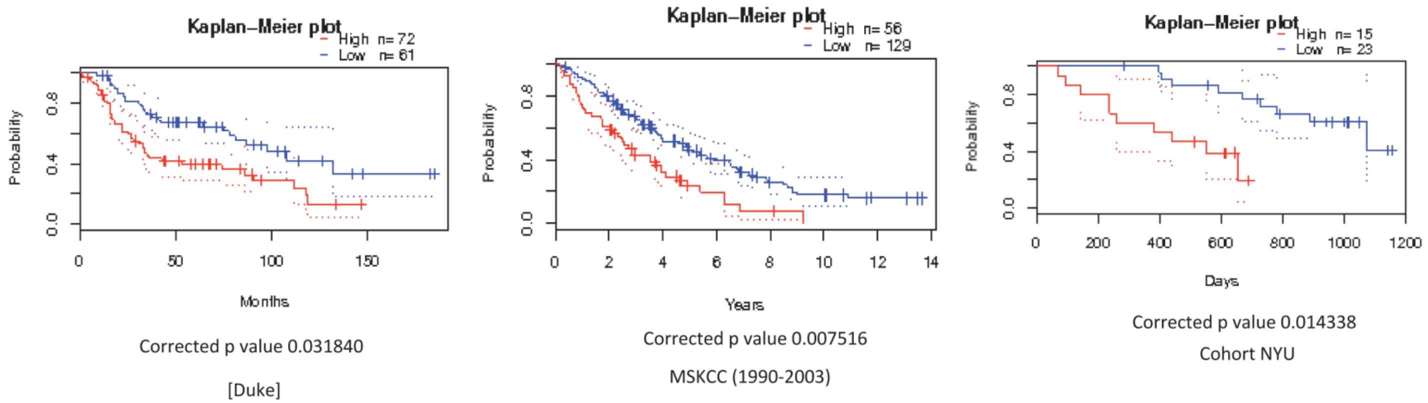

Corrected p value 0.031840

[Duke]

Corrected p value 0.007516

MSKCC (1990-2003)

Corrected p value 0.014338

Cohort NYU

B. DKK2 mRNA expression and survival in epithelial ovarian cancer

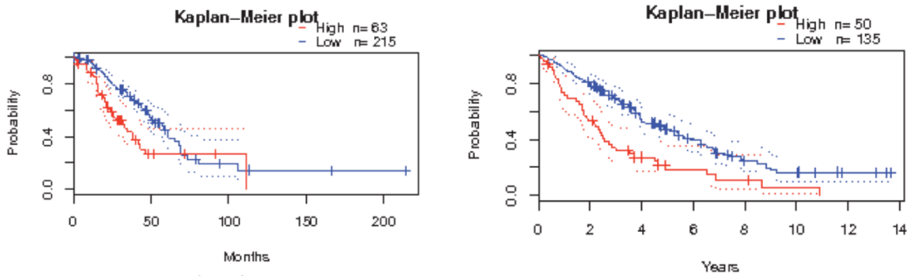

corrected p value 0.007872

AOCS, RBH, WH, NKI-AVL (1992-2006)

corrected p value 0.000773

MSKCC (1990-2003)

Supplementary Figure 3: Kaplan-Meier plots for DKK2 and FZD1 [prognoscan]

Kaplan-Meier plots [PrognoScan] showing decreased overall survival in ovarian cancer patients with up-regulated mRNA expression of the Wnt receptor FZD1 (A) and Wnt feedback component DKK2 (B)

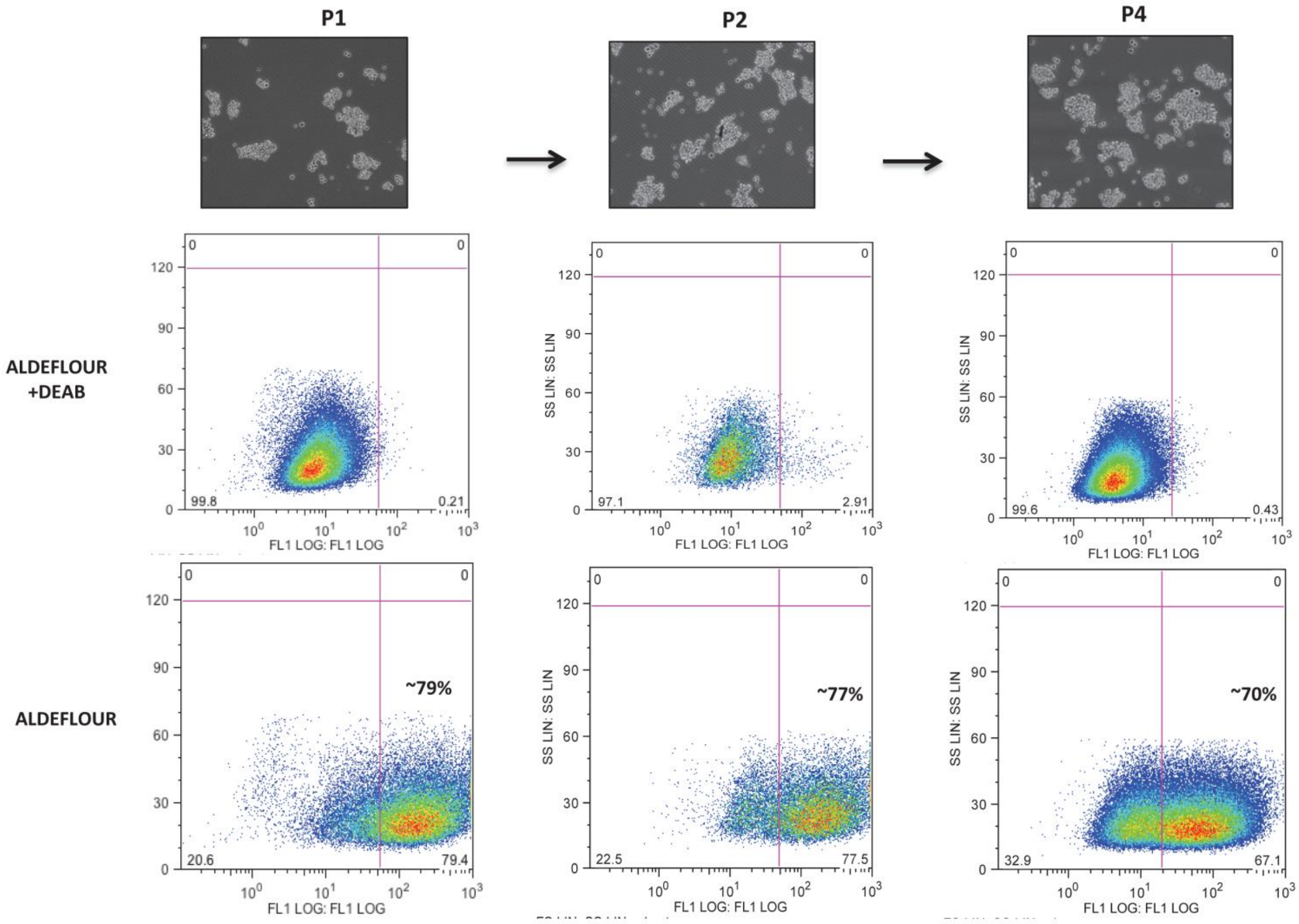

Supplementary Figure 4: ALDH^{pos}CP70 cells maintain tumor sphere formation and ALDH1 activity across passages

10x light microscopy images and ALDEFLOUR activity flow cytometry assay showing that ALDH^{pos} CP70 cells maintain tumor sphere formation ability and ALDH1 activity in non-adherent culture conditions across passages.
